# GSK3β-mediated NRF2 degradation drives *Escherichia coli*-induced ferroptosis in the bovine endometrium

**DOI:** 10.1186/s13567-025-01675-w

**Published:** 2025-12-18

**Authors:** Changning Yuan, Pengfei Dong, Jue Wang, Peng Mao, Zhihao Wang, Junsheng Dong, Long Guo, Kangjun Liu, Luying Cui, Jianji Li, Heng Wang

**Affiliations:** 1 College of Veterinary Medicine, Yangzhou University, Jiangsu Coinnovation Center for Prevention and Control of Important Animal Infectious Diseases and Zoonoses, Yangzhou, 225009 China; 2 International Research Laboratory of Prevention and Control of Important Animal Infectious Diseases and Zoonotic Diseases of Jiangsu Higher Education Institutions, Yangzhou University, Yangzhou, 225009 China; 3 Pancreatic Center, Department of Gastroenterology, Yangzhou Key Laboratory of Pancreatic Disease, The Affiliated Hospital of Yangzhou University, Yangzhou University, Yangzhou, 225000 China

**Keywords:** Bovine, endometritis, *Escherichia coli*, ferroptosis, GSK-3β, NRF2

## Abstract

The infection of the bovine endometrium by *Escherichia coli* often results in endometrial injury with lower fertility in the bovine industry. However, what functions does ferroptosis play during endometritis? This is not completely explained. Therefore, this study aimed to elucidate the potential mechanism of ferroptosis during endometrial injury induced by *E. coli*. The endometrial tissues from sixteen Holstein cows with *E. coli* infection were harvested for pathological examination and RNA-seq. Primary bovine endometrial epithelial cells (BEECs) were used to establish an *E. coli* infection model in vitro. The mechanism by which ferroptosis is involved in endometritis was explored by western blot, flow cytometry, and immunofluorescence methods. The results revealed that the injury of endometrial tissues and BEECs induced by *E. coli* was associated with ferroptosis via Fe^2+^ accumulation, increased lipid peroxidation, decreased glutathione concentration, and inhibited expression of solute carrier family 7-member 11, glutathione peroxidase 4, and ferritin heavy chain 1. Ferroptosis inhibition alleviated BEEC damage induced by *E. coli*. Further analysis revealed that the *E. coli*-induced ferroptosis was associated with impaired nuclear factor erythroid 2-related factor 2 (NRF2) antioxidant defense, which is characterized by mitigated NRF2 degradation and blunted induction of target antioxidant enzymes, such as NAD(P)H quinone oxidoreductase 1. In contrast, promoting the expression of NRF2 mitigated the cellular damage and ferroptosis induced by *E. coli*. In particular, glycogen synthase kinase (GSK)-3β exhibited sustained expression and hyperactivity in endometrial tissue and BEECs infected with *E. coli*, which confirmed that the suppression of GSK-3β could significantly inhibit NRF2 degradation and attenuate ferroptosis. This study demonstrated that *E. coli* can trigger ferroptosis in endometrial tissues and BEECs through GSK-3β-mediated NRF2 degradation. Modulating GSK-3β/NRF2 holds promise as a potential therapeutic strategy for alleviating endometritis induced by *E. coli*.

**Supplementary Information:**

The online version contains supplementary material available at 10.1186/s13567-025-01675-w.

## Introduction

Bacterial invasion and proliferation in the bovine endometrium often induce endometritis, with low fertility and high economic loss in the dairy industry [1]. *Escherichia coli* is a common environmental pathogen that causes bovine endometritis and is capable of significant host cell death and tissue damage [2–4]. While antibiotics are the main treatment for endometritis in dairy cows, their efficacy has been compromised by the increase in drug-resistant strains [5–7]. Consequently, in addition to exploring novel antibiotics, determining the molecular mechanisms underlying endometritis induced by *E. coli* is imperative for the development of innovative adjuvant therapies.

Ferroptosis, characterized by iron accumulation and lipid peroxidation, represents a form of regulated cell death [8]. Some studies highlight the association between ferroptosis and bacterial infections, with ferroptosis inhibition emerging as a promising therapeutic strategy to combat these infections [9, 10]. *Mycobacterium tuberculosis* upregulates the expression of ferroptosis markers, including Fe^2+^ and lipid peroxides, in macrophages [11]. *E. coli* infection suppresses solute carrier family 7-member 11 (SLC7A11)/glutathione peroxidase 4 (GPX4) expression in bovine mammary epithelial cells (BMECs), disrupts iron homeostasis and precipitates ferroptosis [12]. In intestinal epithelial cells (IECs), *E. coli* infection decreases GPX4 and ferritin heavy chain 1 (FTH1) levels and increases lipid peroxidation, thereby inducing ferroptosis [13]. Furthermore, ferroptosis can amplify the inflammatory response through the release of damage-associated molecular patterns (DAMPs) and cytokines, underscoring the substantial anti-inflammatory potential of ferroptosis inhibition [14]. However, the role of ferroptosis in bovine endometritis induced by *E. coli* remains to be fully elucidated.

Nuclear factor erythroid 2-related factor 2 (NRF2), a redox-sensitive transcription factor, plays a pivotal role in mediating the cellular antioxidative stress response [15]. Some studies indicate that NRF2 maintains redox balance to inhibit ferroptosis by modulating the expression of related genes (including GPX4, SLC7A11, NAD(P)H quinone oxidoreductase 1 (NQO1), and the glutamate-cysteine ligase catalytic subunit (GCLC)) [16, 17]. NRF2 enhances the activity of antioxidant enzymes, including superoxide dismutase (SOD), catalase (CAT), and glutathione peroxidase (GSH-Px), to combat bacterium-induced reactive oxygen species (ROS) and malondialdehyde (MDA) [18]. Additionally, NRF2 maintains intracellular iron homeostasis by regulating the expression of FTH1 [19]. Several studies have reported that the inhibition of NRF2 signalling can promote oxidative stress and ferroptosis [20, 21]. However, the specific function of NRF2 in bovine endometritis induced by *E. coli* and the underlying mechanisms of its activity are not fully understood. The classical pathway of NRF2 degradation is mediated by kelch-like Ech-associated protein 1 (KEAP1) [22]. Nonetheless, the ability of glycogen synthase kinase (GSK)-3β to facilitate NRF2 proteasomal degradation through a KEAP1-independent mechanism has been recognized [23, 24]. Specifically, the phosphorylation of GSK-3β at Ser9 leads to the inactivation of its kinase activity [25]. Lipopolysaccharide (LPS) from *E. coli* has also been reported to inhibit the phosphorylation of GSK-3β at Ser9 in primary bovine endometrial epithelial cells (BEECs) [26]. *Salmonella* infection has been reported to reduce the phosphorylation of GSK-3β (Ser9) in mouse colonic epithelial cells within 6 h post-infection [27]. Inhibiting the phosphorylation of GSK-3β (Ser9) in hepatocytes hastens NRF2 degradation [28]. Hence, we propose that *E. coli* might trigger ferroptosis via the GSK-3β-mediated degradation of NRF2 in the bovine endometrium.

This study investigates the underlying mechanisms of ferroptosis in endometrial injury induced by *E. coli* and elucidates the involvement of ferroptosis in endometrial tissues and BEEC damage induced by *E. coli*, highlighting the role of GSK-3β-mediated NRF2 degradation in endometrial ferroptosis induced by *E. coli*. These findings elucidate the mechanism underlying bovine endometrial ferroptosis induced by *E. coli* and advance the development of innovative ferroptosis-targeting therapies for endometritis.

## Materials and methods

### Reagents

The antibodies used in the study were as follows: anti-KEAP1 (#AF5266, Affinity Biosciences, Changzhou, China), anti-NQO1 (#DF6437, Affinity Biosciences), anti-GSK-3β (#AF5016, Affinity Biosciences), anti-GCLC (#DF8550, Affinity Biosciences), anti-β-actin (#AF7018, Affinity Biosciences), anti-PTGS2 (#AF7003, Affinity Biosciences), anti-Lamin B1 (#AF5161, Affinity Biosciences), goat anti-rabbit IgG H&L (#S0001, Affinity Biosciences), goat anti-mouse IgG H&L (#S0002, Affinity Biosciences), mouse anti-rabbit IgG light chain-specific (#A25022, Abbkine, Wuhan, China), anti-FTH1 (#p02794, Abmart Shanghai, China), anti-GPX4 (#A21440, ABclonal, Wuhan, China), anti-NRF2 (#16396-1-AP, Proteintech, Wuhan, China), anti-p-GSK-3β (Ser9) (#sc-373800, Santa Cruz Biotechnology, TX, US), anti-SLC7A11 (#YT8130, Immunoway, CA, USA), and anti-4-Hydroxynonenal (4-HNE; #ab46545, Abcam Cambridge, UK). The following reagents were obtained from MedChemExpress (Shanghai, China): Fer-1 (#HY-100579) and tert-butylhydroquinone (TBHQ) (#HY-100489). Lithium chloride (LiCl) (#L9650) and polyvinylidene difluoride (PVDF) were obtained from Merck (Darmstadt, Germany).

### Animals

Endometrial tissue was sourced from the Holstein dairy cow farm of Yangzhou University, which has a well-established management system. The uteri of healthy dairy cows (35–50 days post-partum) were selected to serve as the control group (*n* = 8). The endometritis group (*n* = 8) consisted of culled dairy cows (35–50 days post-partum) on the farm that exhibited endometritis and were singly infected with *E. coli*. This diagnosis was confirmed through visual examination, rectal palpation, and endometrial cytology [29], and the cows presented symptoms characteristic of endometritis. Uterine secretions were harvested using the cytobrush technique and inoculated in MacConkey agar medium (#HBPM016, Hopebio, Qingdao China). Individual colonies were identified as *E. coli* using the MALDI Biotyper system (Bruker, Karlsruhe, Germany). The endometrial tissue was collected and preserved at −80 °C or 4% paraformaldehyde for subsequent analysis. This study was conducted in accordance with Yangzhou University regulations on animal care and use in research and the ARRIVE guidelines [30].

### Histopathological analysis

Endometrial tissue was fixed in 4% paraformaldehyde solution, embedded in paraffin blocks, and cut into 4-μm sections using an automated microtome (Leica, Wetzlar, Germany). Afterward, the slices were deparaffinized by alcohol with serial dilutions and xylene and stained with hematoxylin and eosin, after which they were examined and scanned under a light microscope (Olympus, Tokyo, Japan). On the basis of the scoring criteria of the endometrial histopathological exam, histopathological changes were assessed using a semiquantitative scale ranging from 1 to 26 [29]. For each section, three fields were randomly selected and evaluated, and the average score was calculated.

### RNA sequencing and protein‒protein interaction (PPI) network analysis

Total RNA from the endometrial tissues were extracted (*n* = 4 per group) using TRIzol reagent (#R701, Vazyme, Nanjing, China). The RNA integrity was verified using an Agilent 2100 Bioanalyzer (Agilent Technologies, USA), with all the samples achieving a RIN > 8.0. After identification with an Agilent 2100 bioanalyzer, the identified RNA was used to construct an RNA library with an NEBNext Ultra II RNA Library Prep Kit (NEB, USA). Transcriptome sequencing was conducted on the Illumina NovaSeq 6000 platform (150-bp paired-end reads; sequencing depth: 40 million reads per sample; Q30 > 90%). Reads were aligned to the bovine genome (NCBI: GCF_000298355.1) using HISAT2 (v2.2.1; parameters: RNA-strandness RF). Gene counts were quantified by employing the featureCounts tool from Subread v2.0.3. The EdgeR and DESeq2 R packages were used for differential expression analysis. A false discovery rate (FDR) < 0.05 and |log_2_ (fold change) |> 1.5 were considered significant. The results were visualized using the “pheatmap” and “ggplot2” R packages. In the DAVID database (accessed on December 25, 2023), Gene Ontology (GO) and Kyoto Encyclopedia of Genes and Genomes (KEGG) enrichment analyses of differentially expressed genes (DEGs) were performed, and *p* < 0.05 was used as the screening condition to screen the results for significant differences. The results of the KEGG and GO enrichment analyses were visualized using the “ggplot2” R package.

Ferroptosis-related genes were downloaded from the FerrDb V2 database (accessed on March 28, 2024). Oxidative stress-related genes were retrieved from the GeneCards database (accessed on March 28, 2024) using “oxidative stress” as the search term and subsequently downloaded. DEGs were merged with the ferroptosis genes set and oxidative stress genes set described above. The overlapping DEGs, termed Fer-DEGs, were visualized using the “pheatmap” R package. Fer-DEGs were uploaded to the STRING database V12.0 (accessed on March 28, 2024), the selected organism was *Bos taurus*, and the default threshold was a combined score > 0.4 to obtain the PPI networks of Fer-DEGs. PPI networks were visualized in Cytoscape V3.9.1 according to degree centrality ranking.

### Cell culture

BEECs were prepared via established protocols [26] and seeded into 25 cm^2^ cell culture flasks (#TCF012050, JET BIOFIL, China). BEECs were cultured under standard conditions at 37 °C in an atmosphere containing 5% CO_2_ in DMEM/F12 (Gibco, USA) enriched with 15% fetal bovine serum (Gibco), 100 U/mL penicillin and 100 μg/mL streptomycin.

### Bacterial culture

A strain of *E. coli* (serotype O_55_) was isolated from a cow with endometritis and cultured on MacConkey agar media [32]. Single colonies were picked and expanded in Luria–Bertani (LB) liquid media. Growth curves were established by measuring the OD_600_. Bacterial numbers were quantified on the basis of growth curves. For the subsequent experimental treatments, single colonies were picked to maintain the growth phase at the logarithmic phase in LB medium. After that, the *E. coli* was washed three times with PBS and diluted with DMEM/F12 for cell treatment.

### Quantitative real-time PCR

RNA from tissues and cells was extracted using TRIzol reagent and reverse transcribed to cDNA using a reverse transcription kit (#R433, Vazyme, Nanjing China). Quantitative real-time PCR (qPCR) was performed using a dedicated SYBR premix (#Q712, Vazyme, Nanjing China). The primer sequences are listed in Table 1. The relative abundances of the mRNA transcripts were calculated via the 2^−△△Ct^ method, with β-actin used as the internal control.

**Table 1:** **Primer sequences for qPCR amplification.**

Genes	primers (5ʹ → 3ʹ)	Product length (bp)
*β-actin*	F: CATCACCATCGGCAATGAGC R: AGCACCGTGTTGGCGTAGAG	144
*IL6*	F: GCCTTCACTCCATTCGCTGTCTC R: AAGTAGTCTGCCTGGGGTGGTG	117
*PTGS2*	F: TGGTCTGGTGCCTGGTCTGATG R: TGTCTGGAACAACTGCTCATCGC	118
*NRF2*	F: ATGACAAGCTGGCTGAGACT R: GTTCACTGTCAACTGGCTGG	197
*NQO1*	F: GGCTCCATGTACTCTCTGCA R: TCTCCAGGCGTTTCTTCCAT	184
*GCLC*	F: AGACAATGCGATTCAAGCCTCCTC R: AGCACCACGAACACCACATACG	121
*SLC7A11*	F: GCCTTGTCCTACGCTGAACT R: GGCTGCAGGGCGTATAATGA	135
*GPX4*	F: CGCAATGAGGCAAGACTGAC R: AAACTGGTTGCAAGGGAAGG	104
*FTH1*	F: TGAGCAGGTGGAAGCCATCAAAG R: CCAGGGTGTGCTTGTCAAAGAGG	110

### Iron, GSH, and MDA assays

Iron location in the endometrium was detected according to the reagent manufacturer's instructions (#G1249, Solarbio, Beijing, China). The stained sections were examined, and images were captured using a light microscope. The average optical density (AOD) of the immunostained samples was quantified using Fiji software (version 2.11.0) [31].

Endometrial tissues were homogenized after being washed with ice-cold phosphate-buffered saline (PBS), and the cells were lysed using sonication. Lysed tissues and cells were centrifuged at 12 000 rpm for 10 min, and the supernatants were collected for protein concentration determination (#P0011, Beyotime, Shanghai, China) and subsequent analysis. The concentrations of Fe^2+^, GSH, and MDA were determined via the protocols provided with the Fe^2+^ Assay Kit (#ab83366, Abcam, Cambridge, UK), GSH Assay Kit (#G263, DOJINDO, Kumamoto, Japan), and MDA Assay Kit (#M496, DOJINDO, Kumamoto, Japan), respectively. The microplate reader (Synergy HT, Bio Tek, VT, US) was set at 593 nm, 532 nm, and 412 nm for the quantification of Fe^2+^, GSH, and MDA, respectively.

### Immunohistochemistry analysis

Deparaffinized sections were incubated with 3% hydrogen peroxide (H_2_O_2_) for 10 min to quench endogenous peroxidase activity, followed by blocking with goat serum for 1 h to reduce nonspecific binding. Afterward, the sections were incubated with primary antibodies, including 4-HNE (diluted 1:400), GPX4 (diluted 1:200), NRF2 (diluted 1:200), and GCLC (diluted 1:200), at 4 °C overnight. The sections were subsequently incubated with the corresponding secondary antibodies at room temperature for 1 h. After color development with 3,3ʹ-diaminobenzidine (DAB) (BOSTER, Wuhan, China), the sections were counterstained with hematoxylin to visualize the cell nuclei. The sections were observed, and images were captured using a light microscope. The AOD of the immunostained samples was quantified via Fiji software for objective analysis of the staining intensity.

### Western blot analysis and co-immunoprecipitation

Total proteins were extracted from tissues and cells using RIPA lysis buffer and PMSF for 30 min on ice. Nuclear proteins were extracted on ice according to the reagent manufacturer’s instructions (#P0028, Beyotime, Shanghai, China). After the protein concentration was adjusted via a bicinchoninic acid (BCA) assay, the same amount of cell lysate was separated via SDS‒PAGE and transferred to PVDF membranes. The membranes were blocked with 5% skim milk for 1 h and then incubated with primary antibodies, including SLC7A11 (1:1000), GPX4 (1:1000), FTH1 (1:1500), PTGS2 (1:1000), NRF2 (1:2000), GCLC (1:1000), NQO1 (1:5000), KEAP1 (1:1000), GSK-3β (1:1000), p-GSK-3β (Ser9) (1:1000), LAMIN B1 (1:1000), and β-actin (1:10 000), separately overnight at 4 °C. Afterward, the membranes were incubated with the secondary antibody (dilution ratio 1:10 000) at room temperature for 1 h. The proteins were visualized with a ChemiScope 5300Pro CCD camera (Clinx, Shanghai, China). All the gray values of the proteins were normalized to those of β-actin via Fiji software. For co-immunoprecipitation (Co-IP), according to the manufacturer’s instructions (Abmart, Shanghai, China), the cell lysate was incubated with anti-NRF2 antibody at 4 ℃ overnight, followed by the addition of protein A/G and further incubation at 4 ℃ overnight. The precipitate was subsequently washed three times with buffer, mixed with 1 × SDS loading buffer and heated at 100 ℃ for 5 min before immunoblotting (IB) analysis.

### Enzyme activity and total antioxidant capacity assays

The endometrial tissues were washed with ice-cold PBS and homogenized, and the BEECs were harvested and lysed via sonication. The supernatants of the lysed tissues and cells were collected by centrifugation for the determination of enzyme activity. Following the manufacturer’s protocols, various enzyme activities were assessed: CAT activity with a Catalase Assay Kit (#A007, Jiancheng, Nanjing, China), GSH-Px activity with a GSH-Px Assay Kit (#A005, Jiancheng), SOD activity with a SOD Assay Kit (#A001, Jiancheng), and GCL activity with a GCL Assay Kit (#A120, Jiancheng). The total antioxidant capacity (T-AOC) was measured using a T-AOC Assay Kit (#A015, Jiancheng).

### Intracellular ROS, lipid peroxidation, and Fe^2+^ detection

BEECs were harvested, and intracellular ROS levels were detected according to the instructions of the ROS assay kit (#S0033, Beyotime). The cells were incubated with dichlorodihydrofluorescein diacetate (DCFH-DA) at 37 ℃ for 30 min. Flow cytometry (CytoFLEX S, Beckman Coulter, CA, USA) was used to observe and record the results at 488 nm.

A total of 6 × 10^5^ cells were seeded in glass-bottom dishes (#BDD012035, BIOFIL, Guangzhou, China), and the cells were challenged with *E. coli* at a multiplicity of infection (MOI) of 10 for 5 h. After treatment, according to the protocol of the BODIPY 581/591 C11 (#L267, DOJINDO, Kumamoto, Japan) and the FerroOrange probe (#F374, DOJINDO, Kumamoto, Japan), the cells were incubated with the respective fluorophores, the BODIPY 581/591 C11 for lipid peroxidation and the FerroOrange probe for the determination of the intracellular iron concentration, at 37 °C for 30 min. The fluorescence was visualized and documented using a live cell workstation (AXIO observer 7, Zeiss, Oberkochen, Germany). The mean fluorescence intensity was quantified using Fiji software for objective analysis of the fluorescence intensity.

### Cell viability assay

Cell viability was assessed via calcein-AM/PI double staining and lactate dehydrogenase (LDH) activity assays. A calcein-AM/PI fluorescence assay was employed to determine the viability of the cells. The BEECs were collected and washed with PBS three times. Then, the cells were incubated with calcein-AM (2 μM) and PI (4.5 μM) for 30 min. Afterward, the cells were flushed with PBS three times again and scanned by a live cell imaging system with excitation at 488 nm for calcein-AM and 561 nm for PI. Cell viability was quantified by analysing 500 calcein-AM-positive or PI-positive cells per sample. The supernatant from the cell culture medium was collected carefully to analyse LDH activity via an LDH activity assay kit (#C0016, Beyotime).

### Colocalization of the GSK-3β and NRF2 proteins in BEECs and endometrial tissues

A total of 2 × 10^5^ BEECs were seeded on cell culture slides, and the cells were challenged with *E. coli* at an MOI of 10 for 5 h. After treatment, the BEECs were fixed with 4% paraformaldehyde for 15 min at room temperature, permeabilized with 0.5% Triton X-100 for 10 min and blocked with 5% bovine serum albumin in PBS for 1 h. Then, the cells were immunostained with antibodies specific to GSK-3β (red, 1:200) and NRF2 (green, 1:100) and counterstained with DAPI (blue) to visualize the cellular nuclei. The colocalization of the GSK-3β and NRF2 proteins was observed via laser confocal microscopy (TCS SP8 STED, Leica) and analysed with Fiji software.

### Analysis of the interactions between NRF2 and GSK-3β

To predict the site of interaction between the NRF2 and GSK-3β proteins, protein‒protein docking was performed according to the described protocol [33]. In brief, on the basis of the amino acid sequences of NRF2 (NP_001011678.2) and GSK-3β (NP_001094780.1), AlphaFold3 was utilized to predict the structure, and the top structure was selected as the subsequent analysis model according to the pLDDT value [34]. The docking model of NRF2-GSK-3β was constructed using the HDOCKlite v1.1 server [35]. The binding free energy of the model with the lowest docking score was subsequently calculated by MM/GBSA of the HawkDock server (parameters: dielectric constant: 80 (solvent)/4 (solute); salt concentration: 0.15 M; implicit solvent model: GBSA (igb = 2); convergence threshold: 0.05 kcal/mol) [36]. The noncovalent interactions at the binding interface of the complex were analysed with the PLIP analysis tool (parameters: hydrogen bonds: distance ≤ 4.1 Å; donor-H-acceptor angle ≥ 120°; hydrophobic interactions: distance ≤ 4.5 Å; salt bridges: distance ≤ 4.0 Å), and the relevant details of the interaction were subsequently supplemented by PyMOL v2.6.

### Effects of LiCl and TBHQ on *E. coli* growth

*E. coli* in the logarithmic growth phase were cultured in DMEM/F12 medium supplemented with 10 mM TBHQ or 20 mM LiCl at 37 °C for 6 h. The optical density (OD) was measured at 600 nm. The bacterial suspension was subsequently serially diluted, cultured and counted until 12 h of incubation at 37 °C for CFU analysis.

### Statistical analysis

SPSS (version 14.0; IL, USA) was used for the analysis. The quantitative data are expressed as the means ± standard errors of the means (SEMs) and were visualized via GraphPad Prism (version 9.5.1; CA, USA). Pearson correlation analysis, which was subsequently conducted to confirm the data's adherence to a normal distribution, was used to quantify the relationship between the histopathological score and NRF2 expression as determined by immunohistochemistry. Unpaired Student’s *t* test was used to compare two groups, and univariate analysis and the Mann‒Whitney U rank-sum test (data not normally distributed) were used for multiple group comparisons. *p* < 0.05 indicated a significant difference between the two groups, and *p* < 0.01 indicated an extremely significant difference between the two groups. All the experiments were repeated three or more times.

## Results

### *E. coli* infection triggers ferroptosis in endometrial tissues

**Figure 1:**
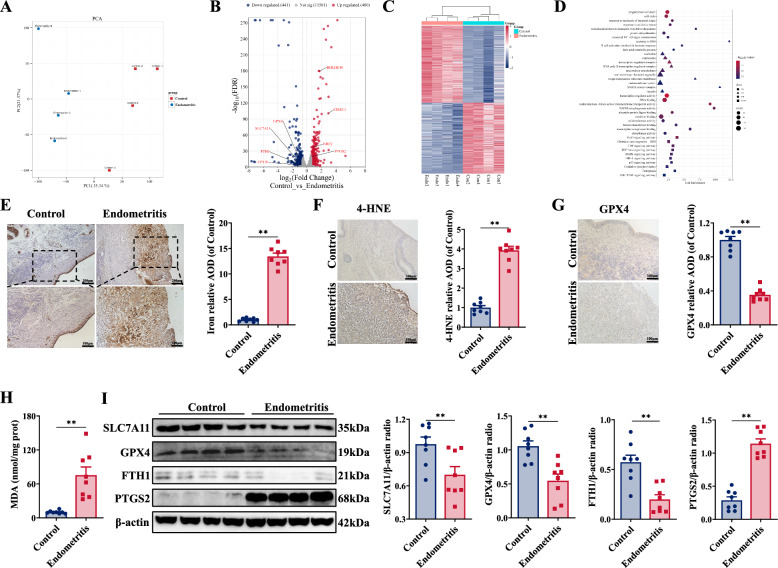
***E. coli* infection caused ferroptosis in the bovine endometrium**. **A** Factor map of the principal component analysis performed on eight tissue samples. Two cluster groups were identified corresponding to the control (red) and *E. coli* infection of the endometrial tissue (blue) (*n* = 4). **B** DEGs were analysed and screened by RNA-seq; red dots indicate genes whose expression is significantly upregulated, green dots indicate genes whose expression is significantly downregulated, and grey dots indicate genes whose expression is not significantly different (*n* = 4). **C** Heatmap of DEGs; red represents upregulated genes, and blue represents downregulated genes (*n* = 4). **D** GO and KEGG enrichment results of the DEGs. **E** Iron concentrations in the endometrium were detected and quantified by Prussian blue staining (*n* = 8). **F** 4-HNE levels in the endometrium were detected and quantified by immunohistochemical staining (*n* = 8). **G** GPX4 expression in the endometrium was detected and quantified by immunohistochemical staining (*n* = 8). **H** The MDA concentration in the endometrium was detected (*n* = 8). **I** SLC7A11, GPX4, FTH1, and PTGS2 expression in the endometrium were detected and quantified by western blotting (*n* = 8). The data are presented as the means ± SEMs. *p < 0.05, **p < 0.01 vs. the control group.

Histopathological examination of the endometrium revealed that endometrial epithelial cells in the endometritis group exhibited irregular arrangement and exfoliation, inflammatory cells infiltration in the matrix layer, increased neovascularization in the matrix layer, and an abundance of inflammatory cells within the uterine glandular cavity (Additional file 1A). Histological scoring indicated significant endometrial injury in the endometritis group (Additional file 1B). RNA-seq was used to investigate the changes in the mRNA transcriptome of endometrial tissues between the endometritis group and the control group, which revealed that 480 genes were upregulated and that 441 genes were downregulated (Figures 1A-C). GO and KEGG pathway enrichment analyses revealed correlations with the oxidative stress response, fatty acid metabolism, and ROS-related pathways, particularly associations with ferroptosis and oxidative stress (Figure 1D). In the endometritis group, more Fe^2+^ was located in the endometrium than in the control group, and the Fe^2+^ concentration also increased significantly (Figure 1E, Additional file 1C). Consistently, the concentrations of 4-HNE and MDA increased, and the concentration of GSH clearly decreased in the endometritis group (Figures 1F, H, Additional file 1D).

Furthermore, the expression of ferroptosis-associated proteins and mRNAs, including SLC7A11, GPX4, and FTH1, was downregulated in endometrial tissues infected with *E. coli* (Figures 1G, I, Additional file 1E), whereas the expression of PTGS2 was upregulated (Figure 1I, Additional file 1M). These results suggest that ferroptosis occurs in the endometrium infected with *E. coli*.

### *E. coli* triggers ferroptosis in BEECs in vitro

**Figure 2:**
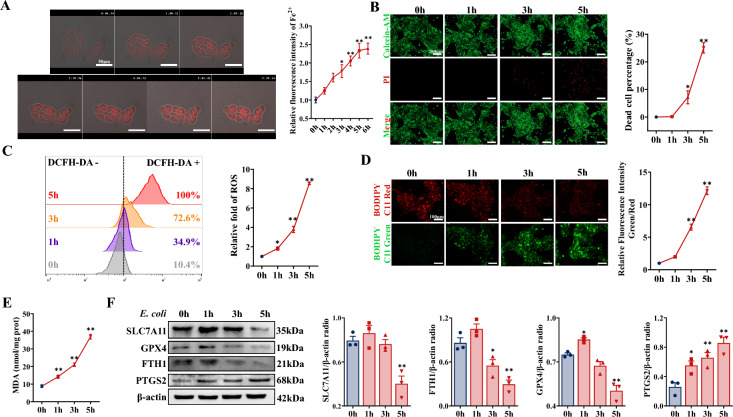
***E. coli* triggered ferroptosis in BEECs in vitro. BEECs were treated with** ***E. coli* (MOI = 10) for 0, 1, 3, or 5 h**. **A** The Fe^2+^ concentration in BEECs was continuously observed and quantified (scale bar, 50 μm) (*n* = 4). **B** Live/dead cells were visualized by calcein-AM/PI staining (scale bar, 200 μm). Dead cells in different groups were counted (*n* = 4). **C** DCFH-DA was used to detect and quantify ROS levels in BEECs (*n* = 3). **D** Lipid peroxidation in BEECs was measured and quantified with BODIPY 583/593 C11 (scale bar, 100 μm) (*n* = 4). **E** The MDA concentration in BEECs was detected (*n* = 4). **F** SLC7A11, GPX4, FTH1, and PTGS2 expression in BEECs were detected and quantified by western blotting (*n* = 3). The data are presented as the means ± SEMs. *p < 0.05, **p < 0.01 vs. the 0 h group.

To delve deeply into the role of ferroptosis in endometritis induced by *E. coli*, BEECs were infected with *E. coli* at an MOI of 10. The results revealed that the Fe^2+^ concentration in BEECs increased, depending on the time of *E. coli* infection, peaking at 5 h (Figure 2A, Additional file 1F, Additional file 2) and that cell damage was exacerbated, as characterized by an increase in the percentage of dead cells and the release of LDH (Figure 2B, Additional file 1G). These changes were accompanied by increases in the levels of ROS, lipid peroxidation, and MDA and a reduction in the concentration of GSH (Figures 2C-E, Additional file 1H). In accordance with the in vitro findings, PTGS2 expression in BEECs increased as the duration of *E. coli* infection increased, whereas GPX4 expression significantly increased at 1 h post-infection, and SLC7A11, GPX4, and FTH1 expression significantly decreased at 5 h post-infection. Although SLC7A11 and FTH1 expression also increased at 1 h post-infection, this increase was not significant (Figure 2F). Collectively, these results suggest that *E. coli* infection contributes to ferroptosis in BEECs.

### Ferroptosis inhibition alleviates BEEC damage induced by* E. coli*

**Figure 3:**
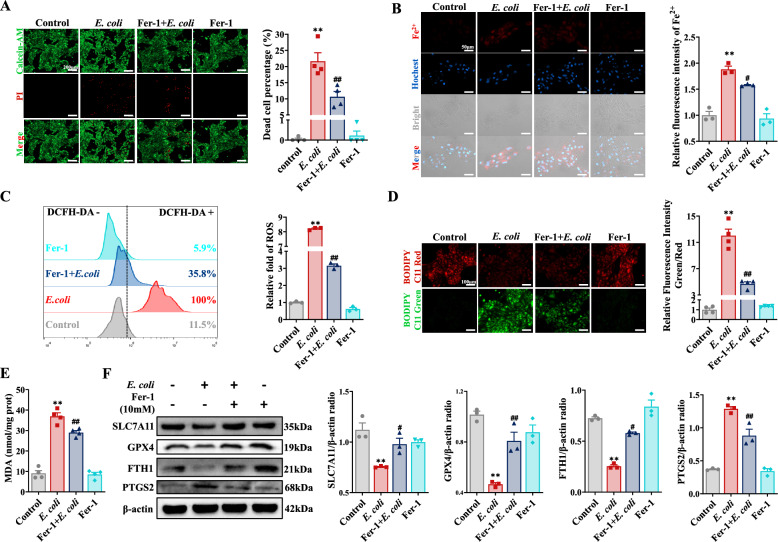
**Fer-1 alleviated** ***E-induced BEEC death. BEECs were pretreated with 10 mM Fer-1 for 2 h and then co-treated with. coli*** ***E. coli* (MOI = 10) for 5 h**. **A** Live/dead cells were visualized by calcein-AM/PI staining (scale bar, 200 μm), and dead cells were counted (*n* = 4). **B** The Fe^2+^ concentration in BEECs was visualized and quantified with a FerroOrange probe (scale bar, 50 μm) (*n* = 3). **C** DCFH-DA was used to detect and quantify ROS levels in BEECs (*n* = 3). **D** Lipid peroxidation in BEECs was measured and quantified with BODIPY 583/593 C11 (scale bar, 100 μm) (*n* = 4). **E** The MDA concentration in BEECs was detected (*n* = 4). **F** SLC7A11, GPX4, FTH1, and PTGS2 expression in BEECs were detected and quantified by western blotting (*n* = 3). The data are presented as the means ± SEMs. *p < 0.05, **p < 0.01 vs. the control group, and ^#^*p* < 0.05, ^##^*p* < 0.01 vs. the *E. coli* group.

To determine the contribution of ferroptosis to BEEC damage induced by *E. coli*, BEECs were treated with Fer-1, an inhibitor of ferroptosis. Calcein-AM/PI double staining and LDH assays demonstrated that the inhibition of ferroptosis significantly mitigated the percentage of dead cells and the release of LDH induced by *E. coli* (Figure 3A, Additional file 1I). Compared with that in the *E. coli* group, the intracellular Fe^2+^ concentration in the Fer-1 + *E. coli* group significantly decreased with the inhibition of ferroptosis (Figure 3B, Additional file 1J). Notably, the increases in ROS, lipid peroxidation, and MDA concentration were also suppressed by Fer-1 treatment, and the decrease in the GSH concentration in BEECs infected with *E. coli* was also reversed (Figures 3C-E, Additional file 1K). Furthermore, the protein expression of GPX4, SLC7A11, and FTH1 increased, whereas PTGS2 protein expression decreased in response to Fer-1 supplementation in BEECs infected with *E. coli* (Figure 3F).

### *E. coli* infection triggers NRF2 degradation

**Figure 4:**
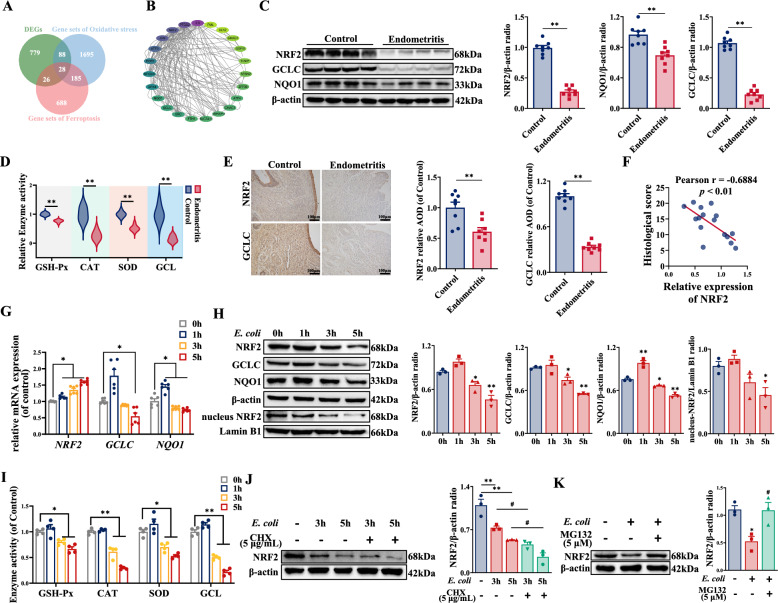
***E. coli* triggered NRF2 degradation**. **A** DEGs were crossed with the ferroptosis-related dataset and the oxidative-stress dataset, and the intersection region was Fer-DEGs. **B** Fer-DEGs were analysed by the PPI network based on the STRING database (accessed on March 28, 2024), which was sorted counterclockwise according to the PPI score; the highest was for *IL6,* and the lowest was for *PML*. **C** NRF2, GCLC, and NQO1 expression in the endometrium were detected and quantified by western blotting (*n* = 8). **D** The enzyme activities of GSH-Px, CAT, SOD, and GCL in the endometrium were detected via kits (*n* = 8). **E** NRF2 and GCLC expression in the endometrium were detected and quantified by immunohistochemical staining (*n* = 8). **F** The correlation between the relative levels of NRF2 in the endometrium and histological scores was analysed by Pearson analysis (*n* = 8). BEECs were treated with *E. coli* (MOI = 10) for 0, 1, 3, or 5 h. **G** The mRNA expression of *NRF2*, *GCLC*, and *NQO1* in BEECs were detected by qPCR (*n* = 6). **H** The expression levels of NRF2, GCLC, and NQO1 in BEECs were detected and quantified by western blotting (*n* = 3). **I** The enzyme activities of GSH-Px, CAT, SOD, and GCL in BEECs were detected (*n* = 4). **J** The cells were cotreated with *E. coli* (MOI = 10) for 3 h or 5 h in the presence or absence of CHX, and NRF2 expression in BEECs was detected and quantified by western blotting (*n* = 3). **K** The cells were cotreated with *E. coli* (MOI = 10) for 5 h in the presence or absence of MG132, and NRF2 expression in BEECs was detected and quantified by western blotting (*n* = 3). The data are presented as the means ± SEMs. *p < 0.05, **p < 0.01 vs. the control/0 h group, and ^#^*p* < 0.05, ^##^*p* < 0.01 vs. the *E. coli* group.

Oxidative stress is intricately linked to ferroptosis, a form of regulated cell death. To elucidate the underlying mechanisms of ferroptosis in BEECs induced by *E. coli*, twenty-eight DEGs related to ferroptosis/oxidative stress (Fer-DEGs) were screened (Figure 4A, Additional file 1L). The Fer-DEGs were subjected to protein–protein interaction (PPI) network analysis with the STRING database (Figure 4B). On the basis of the protein‒protein interaction scores, *IL6*, *PTGS2*, and *NRF2* were identified as crucial hub genes whose expression was significantly upregulated in endometrial tissue infected with *E. coli* (Additional file 1M). IL6 is a regulator of inflammation [37], whereas PTGS2 acts as a marker of ferroptosis [38], and NRF2, a central regulator of the antioxidant response, is essential for maintaining the cellular redox balance. This study highlights the potential significance of NRF2 signalling in mediating ferroptosis induced by *E. coli*. The expression of the NRF2 protein was significantly downregulated in endometrial tissue infected with *E. coli* (Figure 4C), a pattern that differed from the gene expression trend. The expression of both the GCLC and NQO1 genes and proteins also decreased in the endometritis group (Figures 4C, E, Additional file 1N). Furthermore, the activities of GSH-Px, SOD, CAT, and GCL in the endometrial tissues significantly decreased after *E. coli* infection, as did the T-AOC (Figure 4D, Additional file 1O). The immunohistochemical results also confirmed that the protein expression of NRF2 in the endometritis group was significantly lower than that in the control group (Figure 4E). Correlation analysis revealed an inverse relationship between NRF2 expression in endometrial tissue and the histological score (Figure 4F). Changes in NRF2 signalling in BEECs infected with *E. coli* were further assessed in vitro. The mRNA expression of NRF2 increased as the duration of *E. coli* infection increased (Figure 4G). Conversely, NRF2 protein expression was downregulated at 3 h and 5 h post-infection (Figure 4H). The cellular distribution of the NRF2 protein following *E. coli* infection was examined. The results revealed that nuclear NRF2 protein expression was significantly reduced (Figure 4H). The mRNA and protein expression of NQO1 and GCLC decreased at 5 h post-infection (Figures 4G, H). The activities of GSH-Px, SOD, CAT, GCL, and T-AOC were significantly reduced at 3 h and 5 h post-infection, as shown in Figure 4I and Additional file 1P. Given the discordance between NRF2 mRNA and protein expression profiles post-infection, it was hypothesized that *E. coli* infection might facilitate NRF2 degradation. To test this hypothesis, the protein synthesis inhibitor cycloheximide (CHX) and the proteasome inhibitor MG132 were used to assess NRF2 degradation at various infection times on the basis of the concentrations used in a previous study [26]. Compared with *E. coli* alone, CHX intensified the downregulation of NRF2 expression (Figure 4J). However, MG132 reversed the increase in NRF2 expression (Figure 4K). The above results demonstrated that infection with *E. coli* triggered the proteasomal degradation of NRF2, which was concomitant with a reduction in antioxidant enzyme activity.

### NRF2 activation suppresses the ferroptosis of BEECs induced by *E. coli*

**Figure 5:**
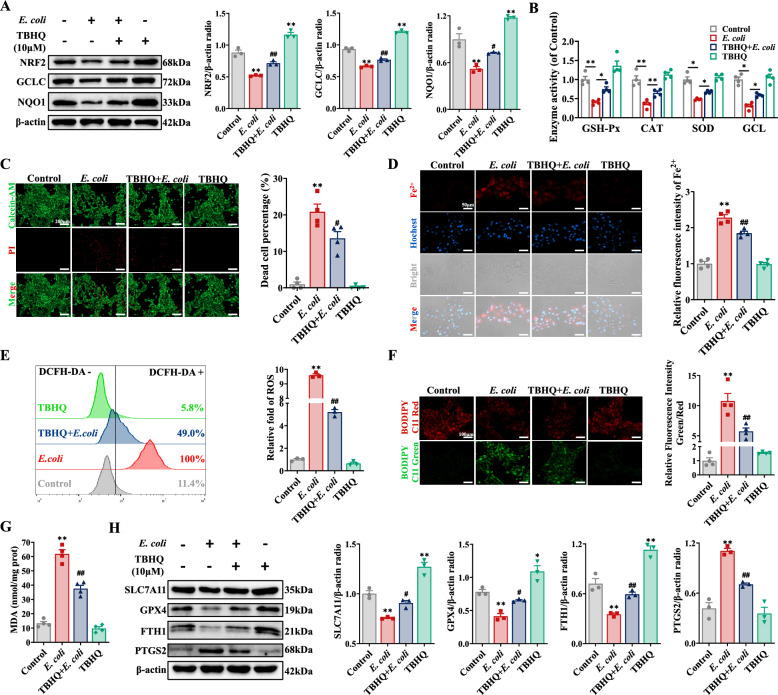
**Activation of NRF2 suppressed** ***E-induced BEEC ferroptosis. BEECs were pretreated with 10 μM TBHQ for 6 h and then co-treated with. coli*** ***E. coli* (MOI = 10) for 5 h**. **A** NRF2, GCLC, and NQO1 expression in BEECs were detected and quantified by western blotting (*n* = 3). **B** The enzyme activities of GSH-Px, CAT, SOD, and GCL in BEECs were detected (*n* = 4). **C** Live/dead cells were visualized via calcein-AM/PI staining (scale bar, 200 μm), and dead cells were counted (*n* = 4). **D** The Fe^2+^ concentration in BEECs was visualized and quantified with a FerroOrange probe (scale bar, 50 μm) (*n* = 4). **E** DCFH-DA was used to detect and quantify ROS levels in BEECs (*n* = 3). **F** Lipid peroxidation in BEECs was measured and quantified with BODIPY 583/593 C11 (scale bar, 100 μm) (*n* = 4). **G** The MDA concentration in BEECs was detected (*n* = 4). **H** SLC7A11, GPX4, FTH1, and PTGS2 expression in BEECs were detected and quantified by western blotting (*n* = 3). The data are presented as the means ± SEMs. *p < 0.05, **p < 0.01 vs. the control group, and ^#^*p* < 0.05, ^##^*p* < 0.01 vs. the *E. coli* group.

To elucidate the relationship between NRF2 and ferroptosis in BEECs infected with *E. coli*, BEECs were pretreated with TBHQ, a specific activator of NRF2. TBHQ notably increased the expression of NRF2, GCLC, and NQO1 in BEECs infected with *E. coli* (Figure 5A), which indicated that the NRF2 signalling pathway was activated in BEECs via TBHQ. Compared with the *E. coli* group, TBHQ also increased the activities of GSH-Px, SOD, CAT, and GCL, as well as T-AOC, in BEECs (Figure 5B, Additional file 3A). Calcein-AM/PI double staining and LDH assays revealed that *E. coli*-induced cell death was significantly decreased by TBHQ (Figure 5C, Additional file 3B). Compared with that in the *E. coli* group, the Fe^2+^ concentration in the TBHQ + *E. coli* group was also significantly lower (Figure 5D, Additional file 3C). Furthermore, the activation of NRF2 with TBHQ significantly suppressed the increase in ROS, lipid peroxidation, and MDA levels and promoted the production of GSH (Figures 5E-G, Additional file 3D). Moreover, TBHQ significantly reversed the changes in SLC7A11, GPX4, FTH1 and PTGS2 expression in BEECs infected with *E. coli* (Figure 5H). Collectively, these findings suggested that the activation of NRF2 by TBHQ suppressed the ferroptosis induced by *E. coli*, thereby mitigating BEEC damage.

### GSK-3β-mediated NRF2 degradation in BEECs induced by *E. coli*

**Figure 6:**
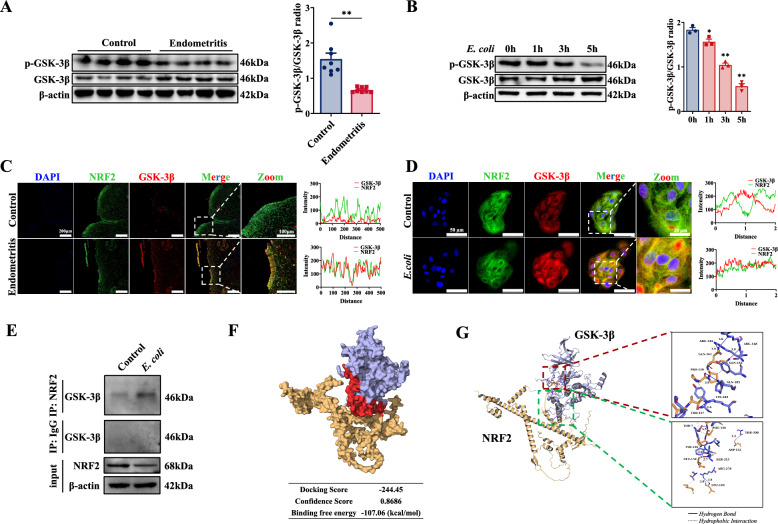
**GSK-3β mediated** ***E-induced NRF2 degradation. coli***. **A** p-GSK-3β and GSK-3β expression in the endometrial tissues were detected and quantified by western blotting (*n* = 8). **B** BEECs were treated with *E. coli* (MOI = 10) for 0, 1, 3, or 5 h, and p-GSK-3β and GSK-3β expression in BEECs were detected and quantified by western blotting (*n* = 3). **C** The endometrial tissues were immunostained, and colocalization was analysed with antibodies against GSK-3β and NRF2. **D** BEECs were treated with *E. coli* (MOI = 10) for 5 h and subsequently fixed for immunostaining and colocalization analysis with antibodies against GSK-3β and NRF2. **E** The cell lysates were subjected to immunoprecipitation with an anti-NRF2 antibody, followed by immunoblot analysis with an anti-GSK-3β antibody. Proteins in whole-cell lysates were used as positive controls (input). **F** Surface representation of the interaction of the NRF2-GSK-3β complex, where the yellow color represents NRF2, the purple color represents GSK-3β, and the red color indicates the binding site of the complex. **G** Interactions between NRF2 and GSK-3β. The lines (blue) represent hydrogen bonds between NRF2 and GSK-3β, and the dotted lines (gray) represent hydrophobic interactions between NRF2 and GSK-3β. The data are presented as the means ± SEMs. *p < 0.05, **p < 0.01 vs. the control group.

The protein expression of KEAP1 was markedly reduced both in vitro and in vivo (Additional files 3E, F), indicating that the NRF2 degradation induced by *E. coli* was likely not mediated by KEAP1. Consequently, the potential role of GSK-3β in NRF2 degradation induced by *E. coli* was investigated. The western blot results revealed a significant reduction in the phosphorylation of GSK-3β at Ser9 (in an inactive state) in endometrial tissue and BEEC *E. coli* (Figures 6A, B). The colocalization of the GSK-3β protein with the NRF2 protein increased significantly in both endometrial tissue and BEECs infected with *E. coli* (Figures 6C, D). Furthermore, Co-IP revealed the interaction between NRF2 and GSK3-β in BEECs infected with *E. coli* (Figure 6E). As shown in Figure 6F, the NRF2-GSK-3β complex model predicted by HDOCKlite had a binding free energy of -107.06 kcal/mol. As shown in Figure. 6G, eleven hydrogen bonds (within 4.1 Å) and multiple sets of hydrophobic interactions formed between the GSK-3β and NRF2 proteins. Hydrogen bonds link Phe156 of NRF2 and Thr7 of GSK-3β, Leu143 of NRF2 and Arg278 of GSK-3β, Asp132 of NRF2 and Thr308 of GSK-3β, Gly152 of NRF2 and Tyr216 of GSK-3β, Glu152 of NRF2 and Ser215 of GSK-3β, Gln161 of NRF2 and Arg144 of GSK-3β, Gln161 of NRF2 and Arg148 of GSK-3β, Pro159 of NRF2 and Gln185 of GSK-3β, Gln163 of NRF2 and Arg148 of GSK-3β, and Thr157 of NRF2 and Lys183 of GSK-3β. Collectively, these results suggest that GSK-3β may mediate the degradation of NRF2 in BEECs infected with *E. coli*.

### Inhibition of GSK-3β alleviates BEEC ferroptosis induced by *E. coli* by blocking NRF2 degradation

**Figure 7:**
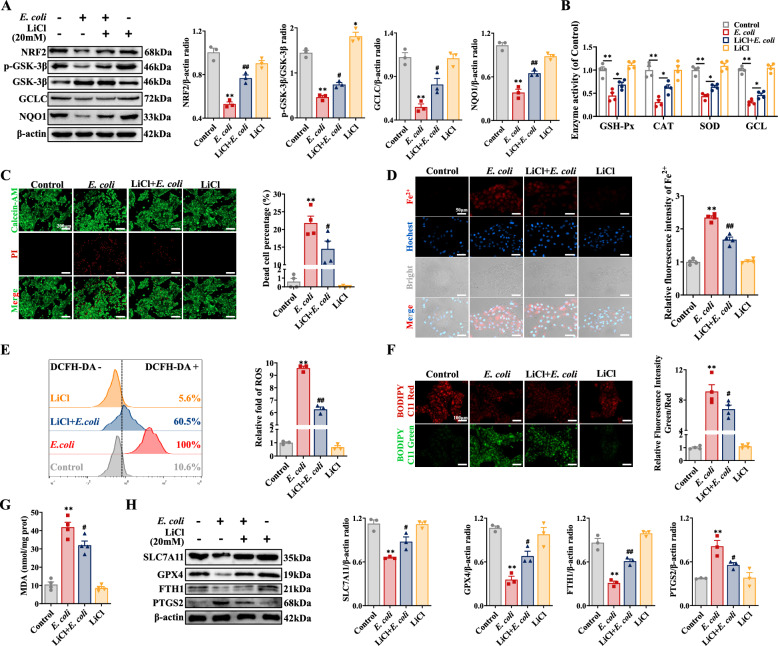
**Inhibition of GSK-3β alleviated** ***E-induced BEEC ferroptosis by blocking NRF2 degradation. BEECs were pretreated with 20 mM LiCl for 6 h and then co-treated with. coli*** ***E. coli* (MOI = 10) for 5 h**. **A** NRF2, p-GSK-3β, GSK-3β, GCLC, and NQO1 expression in BEECs were detected and quantified by western blotting (*n* = 3). **B** The enzyme activities of GSH-Px, CAT, SOD, and GCL in BEECs were detected (*n* = 4). **C** Live/dead cells were visualized via calcein-AM/PI staining (scale bar, 200 μm), and dead cells were counted (*n* = 4). **D** The Fe^2+^ concentration in BEECs was visualized and quantified with a FerroOrange probe (scale bar, 50 μm) (*n* = 4). **E** DCFH-DA was used to detect and quantify ROS levels in BEECs (*n* = 3). **F** Lipid peroxidation in BEECs was measured and quantified with BODIPY 583/593 C11 (scale bar, 100 μm) (*n* = 4). **G** Kits were used to detect the MDA concentration in BEECs (*n* = 4). **H** SLC7A11, GPX4, FTH1, and PTGS2 expression in BEECs were detected and quantified by western blotting (*n* = 3). The data are presented as the means ± SEMs. *p < 0.05, **p < 0.01 vs. the control group, and ^#^*p* < 0.05, ^##^*p* < 0.01 vs. the *E. coli* group.

Lithium chloride (LiCl), a specific inhibitor of GSK-3β, was used to investigate the role of GSK-3β in the ferroptosis of BEECs induced by *E. coli.* The results showed that LiCl significantly increased the phosphorylation of GSK-3β at Ser9 (in an inactive state) and the expression of NRF2, GCLC, and NQO1 in BEECs infected with *E. coli* (Figure 7A). Concurrently, LiCl increased the activities of GSH-Px, SOD, CAT, and GCL in BEECs infected with *E. coli* (Figure 7B), although no significant change in T-AOC was observed (Additional file 3G). These findings indicate that LiCl activated NRF2 signalling by inhibiting GSK-3β in BEECs infected with *E. coli*. Calcein-AM/PI double staining and LDH assays demonstrated that LiCl significantly reduced *E. coli*-induced cell death (Figure 7C, Additional file 3H). LiCl also effectively reversed the elevated Fe^2+^ concentration in BEECs infected with *E. coli* (Figure 7D, Additional file 3I). The inhibition of GSK-3β with LiCl significantly suppressed the increase in ROS, lipid peroxidation, and MDA levels and promoted the production of GSH (Figures 7E-G, Additional file 3J). Western blot analysis revealed that LiCl significantly reversed the altered expression of SLC7A11, GPX4, FTH1, and PTGS2 in BEECs infected with *E. coli* (Figure 7H). Furthermore, this study investigated the direct effects of TBHQ and LiCl on *E. coli* growth. The results revealed that 20 mM LiCl and 10 μM TBHQ did not significantly alter bacterial growth (Additional file 4). Collectively, these results suggested that *E. coli* triggered ferroptosis in BEECs through the modulation of GSK3β-dependent NRF2 degradation and that LiCl inhibited the ferroptosis of BEECs infected with *E. coli.*

## Discussion

The fertility of dairy cows is impaired by endometritis through disruption of endometrial function, creating an adverse environment for embryo production in the female genital tract and affecting ovarian follicle function [39, 40]. Desquamation of endometrial epithelial cells allows bacteria to reach the matrix layer, which leads to injury and inflammation [1]. The damage alleviation of epithelial cells is particularly important for the development of endometritis.

Ferroptosis, characterized by iron dependence and lipid peroxidation-driven cell death, has been increasingly implicated in host tissues infected by bacteria [11, 41]. This study demonstrated that *E. coli* triggered ferroptosis in endometria and BEECs. RNA sequencing revealed significant enrichment of DEGs related to the oxidative stress pathway and ferroptosis pathway, which aligned with the results of the phenotypic observations of endometrial tissue with *E. coli* infection. Specifically, the depletion of GSH, elevation of MDA/4-HNE (end products of lipid peroxidation), and reduced activities of antioxidant enzymes (including SOD, CAT, GCL, and GSH-Px) collectively indicate disrupted redox homeostasis, which is a hallmark of ferroptosis execution. Consistent with a previous report that *E. coli* inhibited SLC7A11/GPX4 signalling in BMECs [12], infection with *E. coli* resulted in the inhibition of SLC7A11 and GPX4 expression in endometrial tissue and BEECs, which may be responsible for the imbalance in redox homeostasis in this study. FTH1 has been reported to buffer iron metabolism by binding free iron [42], while suppression of FTH1 disrupts iron homeostasis and increases lipid peroxidation levels, thereby inducing ferroptosis in IECs infected with *E. coli* [13]. In this study, FTH1 expression was significantly inhibited, resulting in Fe^2+^ accumulation in endometrial tissue and BEECs infected with *E. coli*. The expression of PTGS2, a marker of ferroptosis [38], markedly increased in endometrial tissue after *E. coli* infection. Furthermore, the effects of pretreatment with Fer-1, a commonly used ferroptosis inhibitor, on BEEC damage were evaluated. Fer-1 has been reported to inhibit ferroptosis by reducing excessive ROS production and lipid peroxidation [43]. Fer-1 reversed the extent of lipid peroxidation and the expression of key ferroptosis-associated molecules in BEECs post-infection while alleviating *E. coli*-mediated BEEC damage. These findings indicate that ferroptosis contributes to BEEC damage induced by *E. coli* and that its inhibition could represent a promising therapeutic strategy. However, since the inhibition of ferroptosis did not fully reverse the damage to BEECs induced by *E. coli*, additional cell death pathways, such as apoptosis [44] and pyroptosis [45], may be involved.

NRF2, as a central regulator of oxidative stress, governs the expression of numerous enzymes and proteins involved in lipid peroxidation prevention and iron metabolism modulation. For example, NRF2 enhances the synthesis of GSH by upregulating SLC7A11 and GCLC, which are essential for GPX4 to convert lipid peroxides into lipid alcohols [46]. In mice with mastitis induced by *Staphylococcus aureus*, the expression of NRF2 is significantly inhibited, and the expression of downstream target proteins (including SLC7A11 and GPX4) decreases, leading to ferroptosis and aggravated mastitis [21]. In the present study, the expression of NRF2 was significantly inhibited in endometrial tissue and BEECs infected with *E. coli*. Concurrently, the expression of its downstream targets (including SLC7A11, GCLC, NQO1, and GPX4) was suppressed, antioxidant enzyme activity was decreased, and lipid peroxidation occurred, ultimately triggering ferroptosis. The mRNA expression of FTH1 and synthesis of ferritin are decreased by knockdown of NRF2, thereby disrupting iron homeostasis [20]. In this study, *E. coli*-induced NRF2 inactivation disrupted iron homeostasis by decreasing FTH1 levels and iron reserves, consequently increasing sensitivity to BEEC ferroptosis. TBHQ was reported to activate NRF2 to induce SLC7A11 and GPX4 expression to mitigate LPS-induced ferroptosis in hepatocytes [47]. In this study, TBHQ activated the NRF2 signalling pathway and upregulated the expression of SLC7A11, GCLC, GPX4, NQO1, and FTH1, leading to the recovery of lipid peroxidation and iron concentration to alleviate ferroptosis induced by *E. coli*. Hence, NRF2 plays a significant role in the ferroptosis of BEECs induced by *E. coli*.

In this study, the expression of KEAP1 (a negative regulator of NRF2 [48]) decreased as the duration of *E. coli* infection increased, while the expression of NRF2 was similarly inhibited. These findings suggest that KEAP1-mediated degradation of NRF2 may not be the primary mode of NRF2 degradation induced by *E. coli* infection. The activity of GSK-3β is modulated by its phosphorylation at Ser9. This phosphorylation mimics substrate binding to the site formed by Arg96, Arg180, and Lys205, excluding true substrate proteins and thereby inhibiting GSK-3β activity [25, 49]. Notably, *Yersinia* stimulates the phosphorylation of GSK-3β during the early stages of macrophage infection [50]. LPS has been reported to increase the phosphorylation of GSK3β (Ser9), leading to the degradation of β-catenin in BEECs [26]. In this study, the phosphorylation of GSK-3β (Ser9) decreased as the duration of *E. coli* infection increased, and Co-IP and immunofluorescence revealed the interaction between GSK-3β and NRF2. Inhibition of GSK-3β restored the expression of NRF2 and decreased the activity of antioxidant enzymes in BEECs infected with *E. coli*. Furthermore, the inhibition of GSK-3β reversed the changes in SLC7A11, GPX4, and FTH1 expression, reducing lipid peroxidation and Fe^2+^ accumulation and thereby alleviating the ferroptosis induced by *E. coli*. These results suggested that infection with *E. coli* triggered GSK-3β activation via Ser9 dephosphorylation, promoting the proteasomal degradation of NRF2. NRF2 suppression directly impairs the expression of downstream antioxidants (including SLC7A11, GPX4, GCLC and NQO1), exacerbating lipid peroxidation and Fe^2+^ accumulation (via inhibition of FTH1). This creates a vicious cycle that amplifies ferroptosis. Targeted modulation of the GSK-3β/NRF2 signalling pathway (LiCl for GSK-3β inhibition or TBHQ for NRF2 activation) rescued the oxidative damage and reversed the ferroptosis of BEECs induced by *E. coli*.

While this study provides mechanistic insights into ferroptosis induced by *E. coli*, certain limitations warrant consideration. Although the interaction between GSK-3β and NRF2 has been reported, the specific amino acid residues involved in their binding remain unexplored. In this study, the interaction sites between GSK-3β and NRF2 were predicted. However, further validation through mutagenesis experiments is needed for a comprehensive mechanistic analysis. Although the monolayer of endometrial epithelial cells serves as the first physiological barrier against uterine pathogens and demonstrates physiological relevance, it oversimplifies the multicellular complexity of the intact endometrium. Various studies have shown that ferroptosis efficiently facilitates immune cell defense against bacterial invasion. This model does not fully recapitulate immune‒epithelial crosstalk, which may modulate ferroptosis sensitivity. Nevertheless, in parallel analyses of endometrial tissues (Figures 1 and 4), features of ferroptosis and activation of GSK-3β were consistently demonstrated, validating the pathological relevance of the BEECs findings. Critically, new data confirm that ferroptosis modulators (LiCl and TBHQ) act through host-directed mechanisms without direct antibacterial effects (Additional file 4), further strengthening our conclusion that dysregulation of the GSK-3β/NRF2 axis primarily drives epithelial damage. Future studies using ex vivo endometrial explants or immune-epithelial cocultures will help elucidate the microenvironmental contributions to ferroptosis progression. In addition, while prior studies linked GSK-3β/NRF2 to ferroptosis in noninfected contexts [23, 24], this study revealed a previously unrecognized role in bacterial infection of the endometrium. Although *E. coli* is the primary etiological agent studied here, future work should assess whether GSK-3β/NRF2-driven ferroptosis extends to other endometritis pathogens (e.g., *Trueperella pyogenes*) [1].

**Figure 8:**
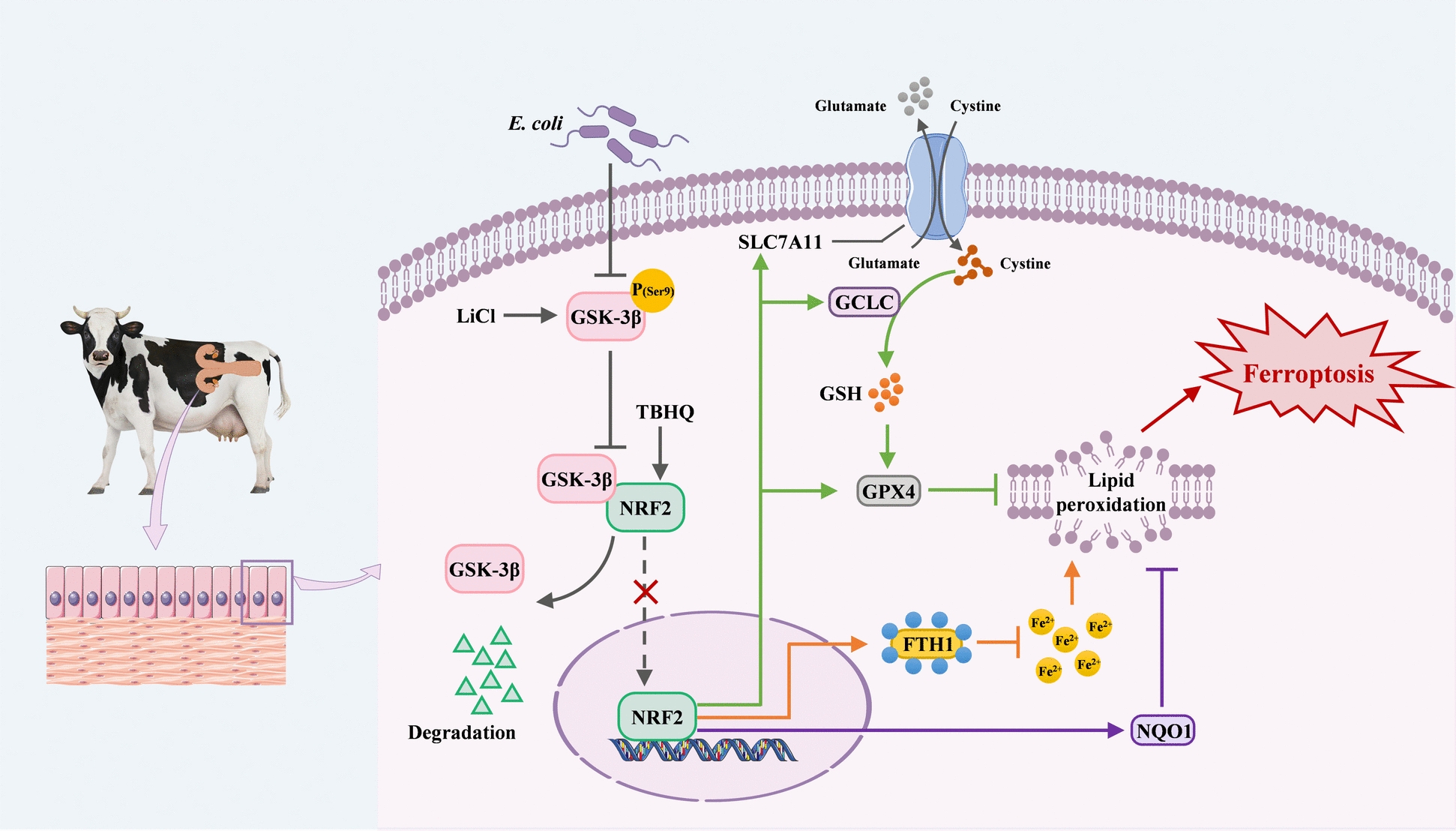
**Potential molecular mechanisms of** ***-induced ferroptosis in BEECsE. coli***.

This study elucidated the involvement of ferroptosis in endometrial injury induced by *E. coli* and its underlying molecular mechanism, highlighting the role of GSK-3β-mediated (not KEAP1-independent) NRF2 degradation in ferroptosis induced by *E. coli*. *E. coli* infection activates GSK-3β through dephosphorylation at Ser9, active GSK-3β binds and degrades NRF2 via the proteasome pathway, and NRF2 suppression disrupts antioxidant defenses (SLC7A11/GPX4 and GCLC/GSH) and iron homeostasis (FTH1), triggering ferroptosis in BEECs (Figure 8). Targeting GSK-3β/NRF2 (e.g., with LiCl and TBHQ) enhances antioxidant capacity, reduces lipid peroxidation levels, and mitigates Fe^2+^ accumulation, thereby inhibiting ferroptosis and protecting host cells. By shifting the therapeutic focus from pathogen eradication to the restoration of epithelial cell tolerance, this promising strategy may transform the management of endometritis caused by infection with *E. coli*.

## Supplementary Information

**Additional file** **1. Ferroptosis involves damage of the endometrium and BEECs infected with** ***E. coli***. **A E-infected endometrial tissues were visualized by H&E staining (scale bar, 100 μm, or 50 μm); red arrows represent disordered epithelial structure and desquamation of epithelial cells, green arrows represent neovascularization, and yellow arrows represent inflammatory cells infiltration.. coli** **B Image scores of H&E-stained endometrial sections from each animal (*n*** **= 8).** ***C* The Fe**^**2+**^** concentration in the endometrial tissues were quantified by a kit (*n = 8).*** **D The GSH concentration in the endometrial tissues were quantified with a kit (*n*** **= 8).** **E** The mRNA expression of SLC7A11, GPX4, and FTH1 in the endometrial tissues were detected by qPCR (*n* = 8). BEECs were treated with *E. coli* (MOI = 10) for 0, 1, 3, or 5 h. **F** The Fe^2+^ concentration was quantified (*n* = 4). **G** LDH enzyme activity in the culture supernatant was quantified. **H** the GSH concentration was quantified. BEECs were pretreated with 10 mM Fer-1 for 2 h and then cotreated with *E. coli* (MOI = 10) for 5 h. **I** LDH enzyme activity in the culture supernatant was quantified via kits. **J** Fe^2+^ concentration was quantified. **K** GSH concentration was quantified (*n* = 4). **L** Heatmap of Fer-DEGs (*n* = 4). **M** mRNA expression of the top 3 Fer-DEGs determined by qPCR (*n* = 8). **N** The mRNA expression of GCLC and NQO1 in the endometrial tissues were detected by qPCR (*n* = 8). **O** The T-AOC of the endometrial tissues was quantified with (*n* = 8). **P** BEECs were treated with *E. coli* (MOI = 10) for 0, 1, 3, or 5 h, and the T-AOC of the BEECs was quantified (*n* = 3). The data are presented as the means ± SEMs. *p < 0.05, **p < 0.01 vs. the control/0 h group, and ^#^*p* < 0.05, ^##^*p* < 0.01 vs. the *E. coli* group.

**Additional file** **2. Dynamic changes in the fluorescence of Fe**^**2+**^

**Additional file** **3**. TBHQ and LiCl alleviated damage induced by *E. coli*. BEECs were pretreated with 10 μM TBHQ for 6 h and then cotreated with *E. coli* (MOI = 10) for 5 h. **A** The T-AOC of BEECs was quantified with a kit (*n* = 4). **B** LDH enzyme activity in the BEEC culture supernatant was quantified with a kit (*n* = 4). **C** The Fe^2+^ concentration in BEECs was quantified via a kit. (*n* = 4). **D** The GSH concentration in BEECs was quantified via a kit. (*n* = 4). **E** KEAP1 expression in the endometrium was detected and quantified via western blotting. (*n* = 8). **F** BEECs were treated with *E. coli* (MOI = 10) for 0, 1, 3, or 5 h, and KEAP1 expression in BEECs was detected and quantified by western blotting (*n* = 4). BEECs were pretreated with 20 mM LiCl for 6 h and then cotreated with *E. coli* (MOI = 10) for 5 h. **G** The T-AOC of BEECs was quantified via a kit. (*n* = 4). **H** LDH enzyme activity in the culture supernatant of BEECs was quantified (*n* = 4). **I** The Fe^2+^ concentration in BEECs was quantified (*n* = 4). **J** The GSH concentration in BEECs was quantified (*n* = 4). The data are presented as the means ± SEMs. *p < 0.05, **p < 0.01 vs. the control/0 h group, and ^#^*p* < 0.05, ^##^*p* < 0.01 vs. the *E. coli* group.

**Additional file** **4**. Effects of TBHQ and LiCl on *E. coli* growth**.** (A&B) Effects of TBHQ on *E. coli* growth (*n* = 10). (C&D) Effects of LiCl on *E. coli* growth (*n* = 10). The data are presented as the means ± SEMs. ns *p* > 0.05 vs. the *E. coli* group.

## Data Availability

The datasets used and/or analysed during the current study are available from the corresponding author upon reasonable request.

## Abbreviations

4-HNE: 4-Hydroxynonenal
AOD: average optical density
BEECs: bovine endometrial epithelial cells
CAT: catalase
CFU: colony-forming unit
CHX: cycloheximide
Co-IP: co-immunoprecipitation
DAB: diaminobenzidine
DAMPs: damage-associated molecular patterns
DCFH-DA: dichlorodihydrofluorescein diacetate
DEGs: differentially expressed genes
*E. coli*: *Escherichia coli*
FDR: false discovery rate
Fer-DEGs: ferroptosis-related differentially expressed genes
FTH1: ferritin heavy chain 1
GCLC: glutamate-cysteine ligase catalytic subunit
GO: Gene Ontology
GPX4: glutathione peroxidase 4
GSH-Px: glutathione peroxidase
GSK-3β: glycogen synthase kinase-3 beta
H_2_O_2_: hydrogen peroxide
IB: immunoblot
IECs: intestinal epithelial cells
KEAP1: kelch-like Ech-associated protein 1
KEGG: Kyoto encyclopedia gene and genome
LB: Luria-Bertani
LDH: lactate dehydrogenase
LiCl: lithium chloride
LPS: lipopolysaccharide
MDA: malondialdehyde
MOI: multiplicity of infection
NQO1: NAD(P)H quinone oxidoreductase 1
NRF2: nuclear factor erythroid 2-related factor 2
OD: optical density
PBS: phosphate-buffered saline
PPI: protein‒protein interaction
PVDF: polyvinylidene difluoride
qPCR: quantitative real-time PCR
RNA-seq: RNA sequencing
ROS: reactive oxygen species
SEM: standard error of the mean
SLC7A11: solute carrier family 7-member 11
SOD: superoxide dismutase
T-AOC: total antioxidant capacity
TBHQ: tert-butylhydroquinone
