# Supplementary figures and images for: GSK3β-mediated NRF2 degradation drives Escherichia coli-induced ferroptosis in the bovine endometrium
Source: Vet Res. 2025 Dec 18;56:232. doi: 10.1186/s13567-025-01675-w (PMC12715911; doi:10.1186/s13567-025-01675-w)

## Matrix layer

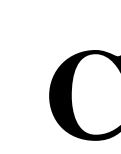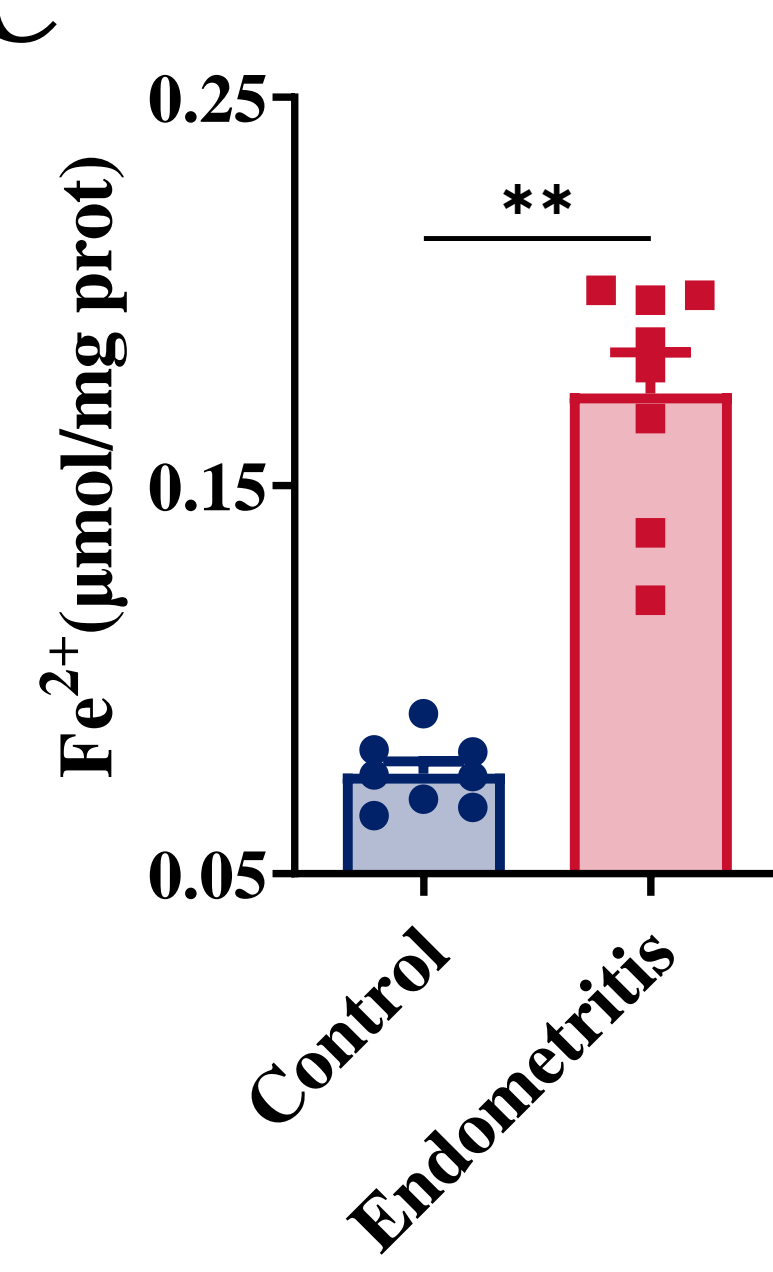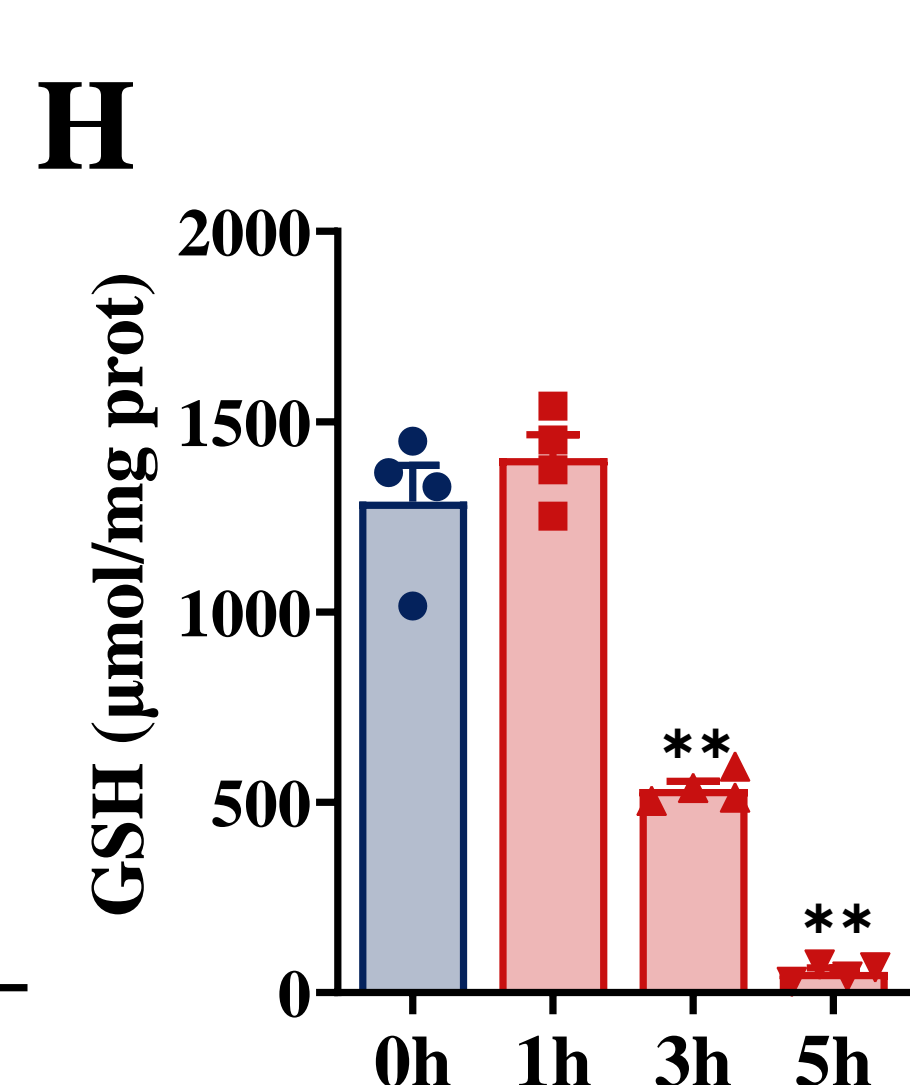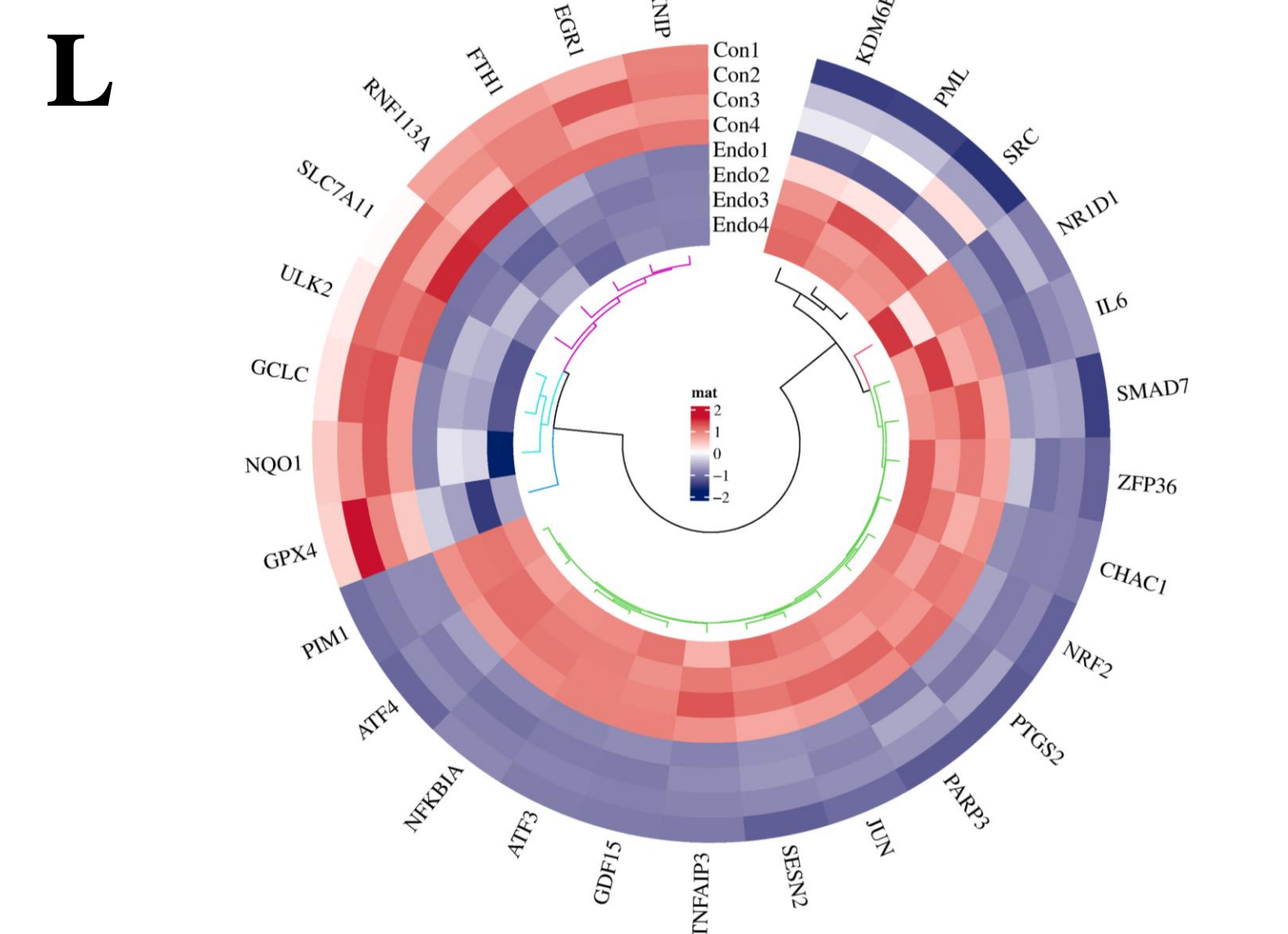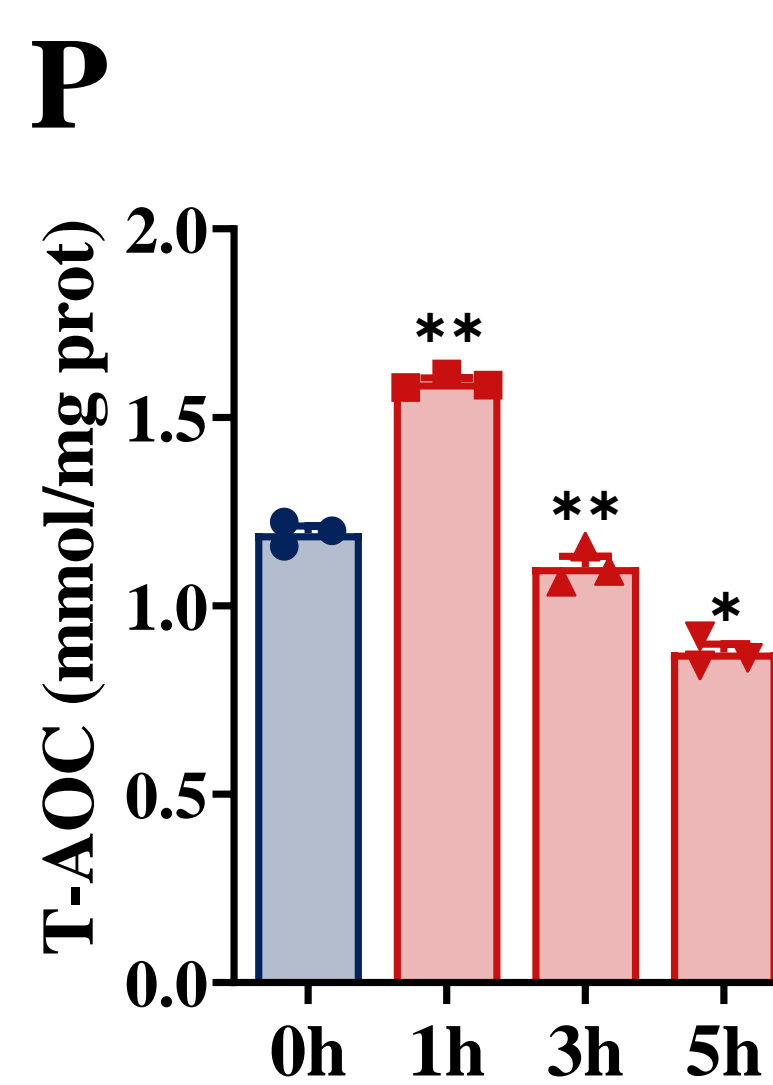

Supplement: Supplementary file 1 — Additional file 1. Ferroptosis involves damage of the endometrium and BEECs infected with E. coli. A E-infected endometrial tissues were visualized by H&E staining (scale bar, 100 μm, or 50 μm); red arrows represent disordered epithelial structure and desquamation of epithelial cells, green arrows represent neovascularization, and yellow arrows represent inflammatory cells infiltration.. coli B Image scores of H&E-stained endometrial sections from each animal (n = 8). C The Fe2+ concentration in the endometrial tissues were quantified by a kit (n = 8). D The GSH concentration in the endometrial tissues were quantified with a kit (n = 8). E The mRNA expression of SLC7A11, GPX4, and FTH1 in the endometrial tissues were detected by qPCR (n = 8). BEECs were treated with E. coli (MOI = 10) for 0, 1, 3, or 5 h. F The Fe2+ concentration was quantified (n = 4). G LDH enzyme activity in the culture supernatant was quantified. H the GSH concentration was quantified. BEECs were pretreated with 10 mM Fer-1 for 2 h and then cotreated with E. coli (MOI = 10) for 5 h. I LDH enzyme activity in the culture supernatant was quantified via kits. J Fe2+ concentration was quantified. K GSH concentration was quantified (n = 4). L Heatmap of Fer-DEGs (n = 4). M mRNA expression of the top 3 Fer-DEGs determined by qPCR (n = 8). N The mRNA expression of GCLC and NQO1 in the endometrial tissues were detected by qPCR (n = 8). O The T-AOC of the endometrial tissues was quantified with (n = 8). P BEECs were treated with E. coli (MOI = 10) for 0, 1, 3, or 5 h, and the T-AOC of the BEECs was quantified (n = 3). The data are presented as the means ± SEMs. *p < 0.05, **p < 0.01 vs. the control/0 h group, and #p < 0.05, ##p < 0.01 vs. the E. coli group. [file 13567_2025_1675_MOESM1_ESM.pdf]

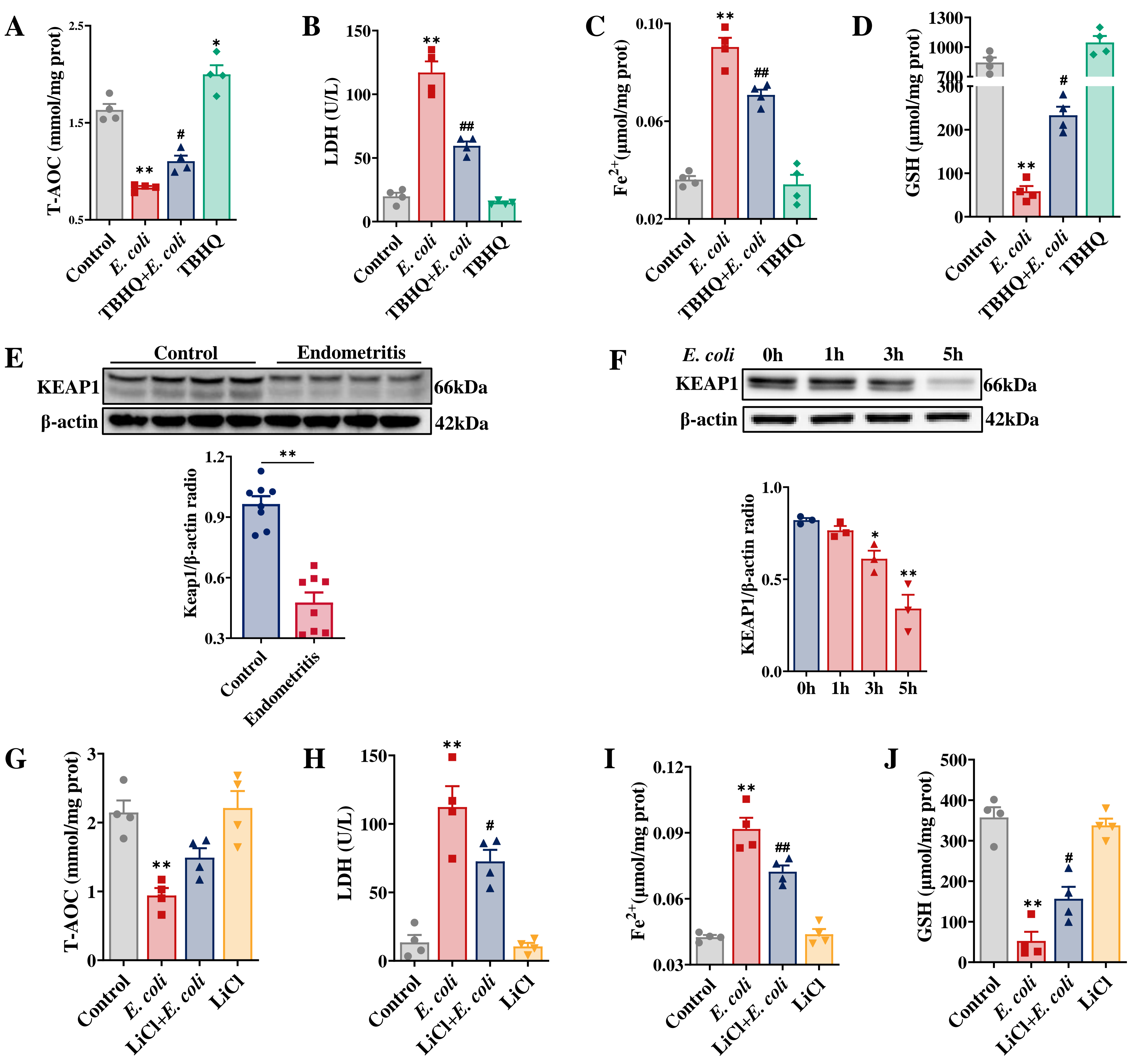

Supplement: Supplementary file 3 — Additional file 3. TBHQ and LiCl alleviated damage induced by E. coli. BEECs were pretreated with 10 μM TBHQ for 6 h and then cotreated with E. coli (MOI = 10) for 5 h. A The T-AOC of BEECs was quantified with a kit (n = 4). B LDH enzyme activity in the BEEC culture supernatant was quantified with a kit (n = 4). C The Fe2+ concentration in BEECs was quantified via a kit. (n = 4). D The GSH concentration in BEECs was quantified via a kit. (n = 4). E KEAP1 expression in the endometrium was detected and quantified via western blotting. (n = 8). F BEECs were treated with E. coli (MOI = 10) for 0, 1, 3, or 5 h, and KEAP1 expression in BEECs was detected and quantified by western blotting (n = 4). BEECs were pretreated with 20 mM LiCl for 6 h and then cotreated with E. coli (MOI = 10) for 5 h. G The T-AOC of BEECs was quantified via a kit. (n = 4). H LDH enzyme activity in the culture supernatant of BEECs was quantified (n = 4). I The Fe2+ concentration in BEECs was quantified (n = 4). J The GSH concentration in BEECs was quantified (n = 4). The data are presented as the means ± SEMs. *p < 0.05, **p < 0.01 vs. the control/0 h group, and #p < 0.05, ##p < 0.01 vs. the E. coli group. [file 13567_2025_1675_MOESM3_ESM.pdf]

**A**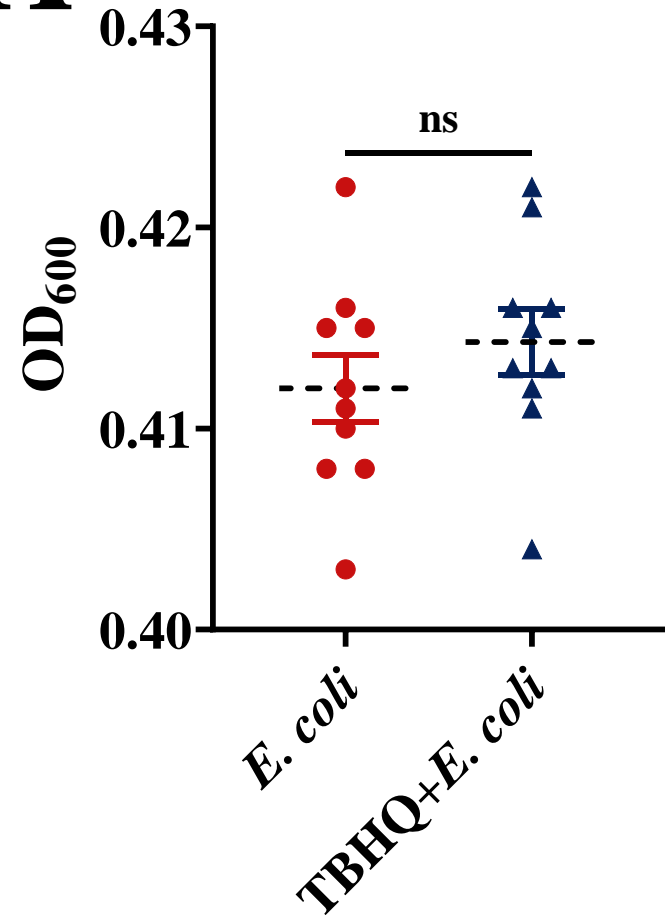**B**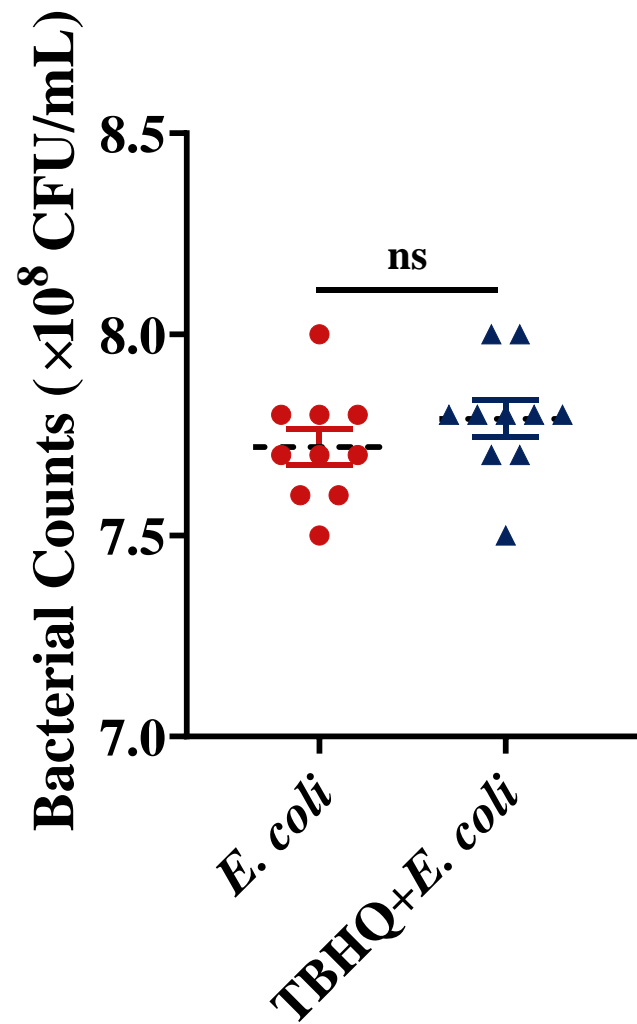**C**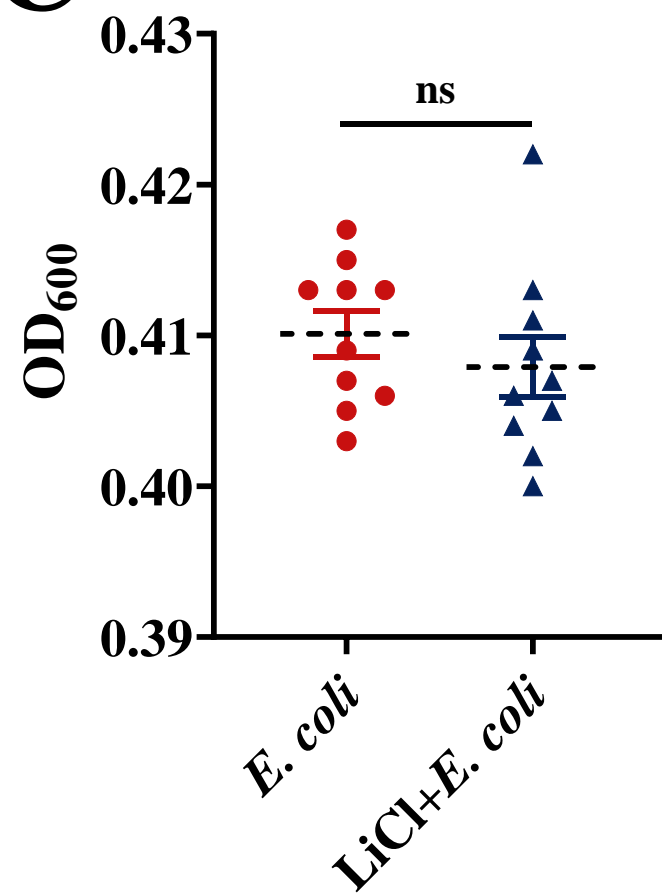**D**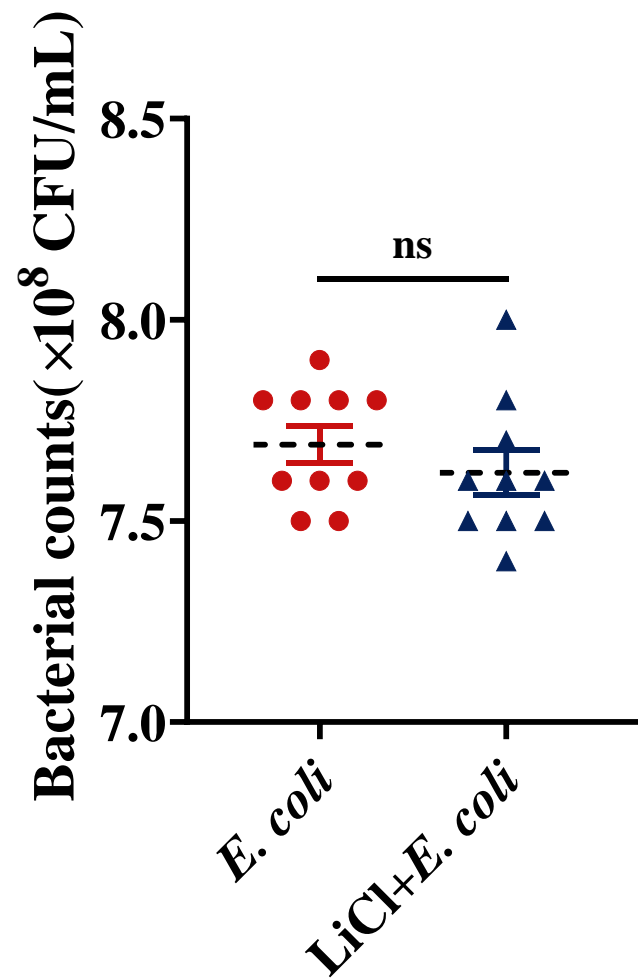

Supplement: Supplementary file 4 — Additional file 4. Effects of TBHQ and LiCl on E. coli growth. (A&B) Effects of TBHQ on E. coli growth (n = 10). (C&D) Effects of LiCl on E. coli growth (n = 10). The data are presented as the means ± SEMs. ns p > 0.05 vs. the E. coli group. [file 13567_2025_1675_MOESM4_ESM.pdf]
